# *Burkholderia pseudomallei* Differentially Regulates Host Innate Immune Response Genes for Intracellular Survival in Lung Epithelial Cells

**DOI:** 10.1371/journal.pntd.0004730

**Published:** 2016-07-01

**Authors:** Kumutha Malar Vellasamy, Vanitha Mariappan, Esaki M. Shankar, Jamuna Vadivelu

**Affiliations:** 1 Department of Medical Microbiology, Faculty of Medicine, University of Malaya, Lembah Pantai, Kuala Lumpur, Malaysia; 2 Tropical Infectious Diseases Research and Education Centre (TIDREC), University of Malaya, Lembah Pantai, Kuala Lumpur, Malaysia; 3 Centre of Excellence for Research in AIDS (CERiA), Wisma R & D, University of Malaya, Lembah Pantai, Kuala Lumpur, Malaysia; University of Tennessee, UNITED STATES

## Abstract

**Background:**

*Burkholderia pseudomallei*, the causative agent of melioidosis poses a serious threat to humankind. *B*. *pseudomallei* secretes numerous virulence proteins that alter host cell functions to escape from intracellular immune sensors. However, the events underlying disease pathogenesis are poorly understood.

**Methods:**

We determined the ability of *B*. *pseudomallei* to invade and survive intracellularly in A549 human lung epithelial cells, and also investigated the early transcriptional responses using an Illumina HumanHT-12 v4 microarray platform, after three hours of exposure to live *B*. *pseudomallei* (BCMS) and its secreted proteins (CCMS).

**Results:**

We found that the ability of *B*. *pseudomallei* to invade and survive intracellularly correlated with increase of multiplicity of infection and duration of contact. Activation of host carbohydrate metabolism and apoptosis as well as suppression of amino acid metabolism and innate immune responses both by live bacteria and its secreted proteins were evident. These early events might be linked to initial activation of host genes directed towards bacterial dissemination from lungs to target organs (via proposed *in vivo* mechanisms) or to escape potential sensing by macrophages.

**Conclusion:**

Understanding the early responses of A549 cells toward *B*. *pseudomallei* infection provide preliminary insights into the likely pathogenesis mechanisms underlying melioidosis, and could contribute to development of novel intervention strategies to combat *B*. *pseudomallei* infections.

## Author Summary

*Burkholderia pseudomallei*, the causative agent of the fatal infectious disease melioidosis, is endemic across parts of South East Asia and Northern Australia. Melioidosis poses a serious worldwide emerging infectious disease problem and bioterrorism threat. Of the key features of *B*. *pseudomallei*, is its ability to remain latent in the host causing recrudescent disease years after initial infection. Relapses are also commonly reported despite appropriate and prolonged antibiotic therapy, suggesting the bacteria’s ability to escape the host’s front-line immune defenses and to manipulate the host’s responses to sustain survival in the host. However, the likely underlying mechanisms of bacterial persistence still remain unclear. Thus, here we proposed to study the host responses towards early interaction of the cell with live *B*. *pseudomallei* and its secretory proteins, in order to understand the potential roles of innate responses against the bacteria.

## Introduction

Intracellular bacteria are known to cause persistent infections and accounts for substantial rates of mortality across the globe each year posing considerable challenge to humankind [1]. These pathogens, including *Burkholderia pseudomallei*, a Gram-negative facultative intracellular pathogen that causes a fatal systemic disease called melioidosis, have evolved distinct strategies to improve their chances of survival and create a safe niche for replication in the host. *B*. *pseudomallei* is predominantly found in the soils of Southeast Asia and Northern Australia [2] and has been characterized as a potential Category B biothreat agent by the Centers for Disease Control and Prevention, USA [3].

*B*. *pseudomallei* is reported to cause acute fulminant pneumonia and septicaemia in endemic areas, and is characterized by multiple abscesses with ~40% mortality rates [4, 5]. Infection is mainly acquired via inhalation and inoculation of the bacteria through breaches in skin [6]. *B*. *pseudomallei* appears to secrete numerous virulence factors, survive and multiply in both phagocytic and non-phagocytic cells as well as escape from membrane-bound phagosome into the cytoplasm after internalization [7, 8]. The ability of *B*. *pseudomallei* to induce cell-to-cell fusion, multinucleated giant cell (MNGC) formation, actin-dependent motility for cell-to-cell spread to evade from host immune surveillance, and escape from autophagy have also been described [9, 10]. Of note, the key feature of the bacteria is its ability to remain latent in the host causing recrudescent disease following years after initial infection [11, 12]. Relapse is quite common despite appropriate antibiotic therapy and presence of high humoral responses [13]. These attributes are suggestive of its ability to evade primary innate defenses and manipulate host responses to sustain survival in the host.

Of the various factors associated with *B*. *pseudomallei*, specialized secretion systems, namely the type 3 (T3SS) and type 6 secretion systems (T6SS) are considered vital to bacterial virulence, owing to their roles in facilitating invasion and intracellular survival in the mammalian host [14]. *The T3SS* effectors of *B*. *pseudomallei* have been shown to enable escape of the bacteria from phagosomes into the cytosol where it could polymerize host actin to render their propulsion throughout the cell. This BimA-dependent intracellular motility allows the bacteria to move efficiently through both the epithelial and macrophage cells while avoiding the host immune responses [15]. The surface polysaccharides of *B*. *pseudomallei* such as the capsular polysaccharides (a major component of Gram-negative cell envelopes) and lipopolysaccharide (LPS or endotoxin) have been reported to inhibit opsonophagocytosis and confer resistance to killing by host complement [16]. However, to date, the mechanisms underlying the ability of *B*. *pseudomallei* to escape from host innate defenses to cause persistent disease still remains ambiguous. Recently, we mapped and profiled the various extracellular proteins of *B*. *pseudomallei* and identified several proteins associated with bacterial virulence [17]. Furthermore, we also postulated that these secretory proteins could play crucial roles in host-pathogen interactions.

To date, the molecular mechanisms that underlie the intracellular lifestyle of *B*. *pseudomallei* remain unclear. Therefore the challenge will be to understand how the bacteria exploit the host responses to be able to successfully replicate and survive within the intracellular compartment. Here, we investigated the host transcriptional responses displayed by A549 human lung epithelial cells resulting from early interaction of the cell with live *B*. *pseudomallei* and its secretory proteins, offering scope to deduce the potential roles of likely innate responses against bacterial invasion.

## Materials and Methods

### Ethical approval

Ethics approval was not required since no human participants were involved in the study. Nonetheless, the study was approved for conduct by the Institutional Biosafety Committee of the University of Malaya.

### Bacterial strains and culture conditions

A haemoculture isolate of *B*. *pseudomallei* (CMS) recovered from clinical septicemic melioidosis at the University of Malaya Medical Centre (UMMC) was used in the current investigation. The clinical isolate was deposited into the bacterial archival collection of the Department of Medical Microbiology, University of Malaya. A non-invasive *Escherichia coli* ATCC 25922 strain was used as a negative control in the investigation. Preparation of the bacterial cultures was performed as previously described [17].

### Preparation of bacterial inoculum for infection

A single colony of *B*. *pseudomallei* from an overnight culture at 37°C was inoculated into 10mL Luria Bertani (LB) broth. The bacteria was cultured aerobically overnight with an agitation of 150rpm at 37°C until OD_600nm_ of 0.8–1.0 was reached. Subsequently, the bacteria were recovered by centrifugation (4000xg for 5mins) with fresh LB and used to inoculate a second liquid culture to obtain an OD600 nm of 0.1. One milliliter of the culture at OD_600nm_ = 0.1 was centrifuged at 4000g for 5mins. The resulting pellet was resuspended in 1mL of RPMI medium and incubated at 37°C for 30mins. Prior to infection of the lung epithelial cell line (A549), the bacterial number was adjusted based on the predetermined growth curve. Following infection, the remaining inoculum was simultaneously plated to reconfirm bacterial count in the inoculum.

### Preparation of bacterial secretory proteins

Bacterial inoculum was prepared as described above. Subsequently, 1mL of the culture with OD_600nm_ of 0.1 was inoculated into 1000mL of LB broth and grown to stationary phase for 20h. The culture was centrifuged at 20000x*g* for 40mins at 4°C and the resulting supernatant was filtered through a 0.22 mm filter (Millipore, USA) to obtain bacteria-free culture supernatant, and concentrated using ultra-filtration as described [20], with minor modifications. Briefly, the culture supernatant was concentrated 20-fold using a Quixstand bench top system (GE Healthcare, Darmstadt, Germany). The supernatant obtained was further concentrated to 50-fold by ultra-filtration employing 10kDa centricon ultra-free centrifugal filter units (Millipore, Massachusetts, USA). The samples were subjected to overnight dialysis using 0.1M phosphate buffered saline (PBS) and the protein concentration was determined using the Bradford method [18].

### Infection of A549 human lung epithelial cells

Infection of A549 cells (ATCC, USA) cells was performed as described [19], with minor modifications. The cells were seeded (5X10^5^ cells/well) into a 24-well culture plate and incubated overnight at 37°C in a 5% CO_2_ incubator. Later, the confluent monolayers were washed three times with PBS to remove dead cells before adding fresh RPMI. The adjusted inoculum was added into wells at a multiplicity of infection (MOI) of 1:10, 1:100 and 1:200. Non-invasive *E*. *coli* was used as negative control.

### Invasion assay

Invasion assays were performed as previously described [19], with slight modifications. Briefly, following infection of A549 cells, the plates were incubated for 1, 2, 3, 6, 12, 18, 24h at 37°C in a 5% CO_2_ environment to facilitate bacterial invasion. Later, the monolayers were washed three times with PBS and 1mL of RMPI containing a cocktail of ceftazidime (1mg/mL) and imipenem (1mg/mL) was added to each well for 2h at 37°C in order to completely eliminate potential residual extracellular bacteria. The cell monolayers were washed three times with PBS and lysed using tergitol solution (0.5% tergitol and 1% BSA in PBS) and serial dilutions of the lysate were plated on nutrient agar (NA) to determine the number of intracellular bacteria [20]. This assay was performed in triplicates of three independent experiments with the results averaged and standard deviation calculated.

### Intracellular survival and replication assay

Intracellular survival assay was performed similar to the invasion assay as described above. Following 2h of incubation with RPMI containing antibiotic to kill the residual extracellular bacteria, the monolayers were washed 3X with PBS. The monolayers were further incubated for 1, 2, 3, 6, 12, 18, 24h in RPMI medium containing ceftazidime (10μg/mL) and imipenem (10μg/ml). The A549 cells were then lysed using tergitol solution (0.5% tergitol and 1% BSA prepared in PBS) and serial dilutions of the lysate were plated onto NA to determine the number of intracellular bacteria [20]. The assay was performed in triplicates with the results averaged and standard deviation calculated.

### Cell viability assay

Cell viability assay was performed as previously described [21], with slight modifications. Briefly, exposure of A549 cells to live *B*. *pseudomallei* or secretory proteins was performed using A549 cells (1X10^6^ cells/mL) seeded in T25 tissue culture flasks. The cells were grown to confluency (1X10^7^ cells/mL) at 37°C in a 5% CO_2_ environment. The monolayers were washed three times with PBS and exposed to live *B*. *pseudomallei* at determined MOIs of 1:10, 1:100 and 1:200, or filter sterilized secretory proteins at concentrations of 0.5, 1, 2, 5, 10, 25, 50 and 100μg/mL. After three hours, the cells were washed with PBS, trypsinized using 0.1% trypsin, collected in RNase-free microfuge tubes and centrifuged at 300g for 5mins. The resulting pellets were washed three times at 300g for 5 mins and subjected to cell viability assay using a 0.4% trypan blue exclusion method [22]. Three replicate flasks containing confluent monolayers were used as biological controls for each of the different MOIs of live bacteria or secretory proteins used. The MOI (1:10) and secreted proteins concentration (5μg/mL) that sustained 95–100% cell viability was selected for the microarray experiment.

### Gene expression

A549 cells exposed for 3 hours to live *B*. *pseudomallei* (MOI 1:10) or secretory proteins (5μg/mL) and control (in triplicates) were trypsinized individually and pelleted by centrifugation at 300x*g* for 5mins. Later, RNA extraction was performed using a commercial RNeasy Mini Kit (Qiagen, USA) according to the manufacturer’s instructions. Concentration and purity of RNA was analysed using a RNA 6000 Nano Bioanalyser (Agilent, USA). cRNA for hybridization on the microarray chip was prepared using the Illumina TotalPrep RNA Amplification Kit (Ambion, USA) according to the manufacturer’s instructions. Microarray analysis was performed using the Whole-Genome Gene Expression Direct Hybridization Assay employing the HumanHT-12 v4 Expression BeadChip (llumina, USA) according to the manufacturer’s instructions. Briefly, the cRNA samples were applied to the arrays on a BeadChip and hybridised at 58°C overnight. Signals developed with streptavidin-Cy3 and the BeadChip were scanned using a Illumina BeadArray Reader (Illumina, USA).

### Microarray analysis

The resulting image of the decoded gene expression data was subjected to further analysis using the GenomeStudio Gene Expression Module (Illumina, USA). The quality of hybridization was determined using internal controls present in the Human HT-12 v4 Expression BeadChip. The raw microarray data was subsequently recovered and subjected to standard normalization procedures for one-colour array data using GeneSpring GX version 11 (Agilent Technologies, USA). The data was normalized by dividing the intensity of each probe by the median intensities for all samples. Subsequently, a box plot was used to check for presence of outliers, and sample hierarchical clustering was performed. One-way ANOVA was used to obtain the number of differentially expressed genes (level of significance, p≤0.05). The data was filtered using the Volcano Plot to obtain the differentially expressed genes with an absolute change >2-fold relative to uninfected controls. Free web-based software was used for further analysis of the genes. The GeneSet Analysis (http://www.bioinfo.vanderbit.edu/) was used to identify Gene Ontology of the differentially expressed genes. The pathways significantly regulated by the genes were also identified using GeneTrail (http://genetrail.bioinf.uni-sb.de/), the Kyoto Encyclopaedia of Genes and Genomes (KEGG) mapper database (http://www.genome.jp/kegg/) and GATHER (http://gather.genome.duke.edu/). The Cluster 3.0 and Java Treeview V1.1.3 softwares were used for hierarchical clustering and visualization of the differentially expressed genes, respectively.

### Validation of microarray data

The microarray data was validated using quantitative real-time PCR (qRT-PCR) analysis using the iQ5 System (Biorad, USA). Ten genes including eight that were significantly regulated in the microarray analysis and two reference genes were used for the validation. *β-actin* and *GAPDH* were used as reference genes for normalization. Primers for the 10 genes identified were selected from a public resource for PCR primers, the PrimerBank (http://pga.mgh.harvard.edu/primerbank/) (Table 1). qRT-PCR was performed (in triplicate) using templates generated from RNAs extracted from independent experiments. Briefly, 25 μL reactions were prepared using primers at a final concentration of 1 μM and the Qiagen One-Step RT-PCR kit with SYBR Green according to the manufacturer’s instructions (BioRad, USA). The thermocycling condition consisted of an initial denaturation for 3 min at 95°C followed by 40 cycles with 15s at 95°C, 30s at 56°C and 30s at 72°C. Fluorescence data was captured at the elongation step of each cycle. Following amplification, melt curves were acquired by increasing the temperature from 65 to 95°C at the rate of 0.5°C 10s^-1^, with continuous measurement of fluorescence. A blank (non-template control) was also incorporated with each assay.

**Table 1:** Primer sequences of the genes used for validation of microarray results using qRT-PCR.

Primer	Sequence 5’– 3’	Amplicon size (bp)
*β-actin*	CAC CTT CAC CGT TCC AGT TT	102
	GAT GAG ATT GGC ATG GCT TT	
*GAPDH*	TGT TGC CAT CAA TGA CCC CTT	102
	CTC CAC GAC GTA CTC AGC G	
*G6PC2*	CAG AAG GAC TAC CGA GCT TAC T	153
	CCA ATC CCC AAT GAC TGC TAC	
*CES1*	CAA GGC GGG GCA GTT ACT C	118
	TTT CTT GGT CAA GTC AGC AGG	
*CXCR7*	TCT GCA TCT CTT CGA CTA CTC A	130
	GTA GAG CAG GAC GCT TTT GTT	
*LAYN*	GCG TGG TCA TGT ACC ATC AG	176
	AGG TGT TGT CAG CTC TGT TTC	
*SERPINA3*	CCT GAA GGC CCC TGA TAA GAA	196
	GCT GGA CTG ATT GAG GGT GC	
*PYCARD*	TGG ATG CTC TGT ACG GGA AG	110
	CCA GGC TGG TGT GAA ACT GAA	
*FXYD*	ATC CTC CTC AGT AAG TGG GGT	101
	CTT GGC AAC TCC CGA AAG C	
*FST*	ACG TGT GAG AAC GTG GAC TG	151
	CAC ATT CAT TGC GGT AGG TTT TC	

## Results

### *B*. *pseudomallei* invades and survives intracellularly within A549 human lung epithelial cells

Invasion of the *B*. *pseudomallei* isolate was found to correlate with the MOI used, and the trend observed was similar at all the different MOIs used (Table 2). The invasion efficiency at 1h post-infection was very low (0.000125–0.0002%) at all the MOIs used. In general, as the post-infection time was increased from 3-12h, a gradual increase in the number of intracellular bacteria was seen at all the MOIs used, reaching a maximum of 3.15 to 3.65% relative to the initial inoculum. This was followed by a gradual decrease between 18 and 24h post-infection, with the percentage of intracellular bacteria relative to the initial inoculum ranging from 1.82 to 2% 24h post-infection. The non-invasive *E*. *coli*, which was used as a negative control, did not show any invasion into the intracellular compartment.

**Table 2:** Mean percentage of invasion (%) with standard deviation MOI 1:10, 1:100 and 1:200.

Hours	% Invasion (MOI 1:10)	% Invasion (MOI 1:100)	% Invasion (MOI 1:200)
**1**	0.000125±0.00004	0.000168±0.00002	0.0002±0.00011
**2**	0.00232±0.00100	0.00322±0.00085	0.0048±0.00093
**3**	0.385±0.01400	0.399±0.01400	0.535±0.01200
**6**	1.725±0.05500	1.888±0.02300	1.987±0.09900
**12**	3.15±0.020000	3.28±0.09000	3.65±0.05200
**18**	2.08±0.070000	2.00±0.05000	2.20±0.05000
**24**	1.83±0.20000	1.75±0.15000	1.82±0.07500

**Fig 1:**
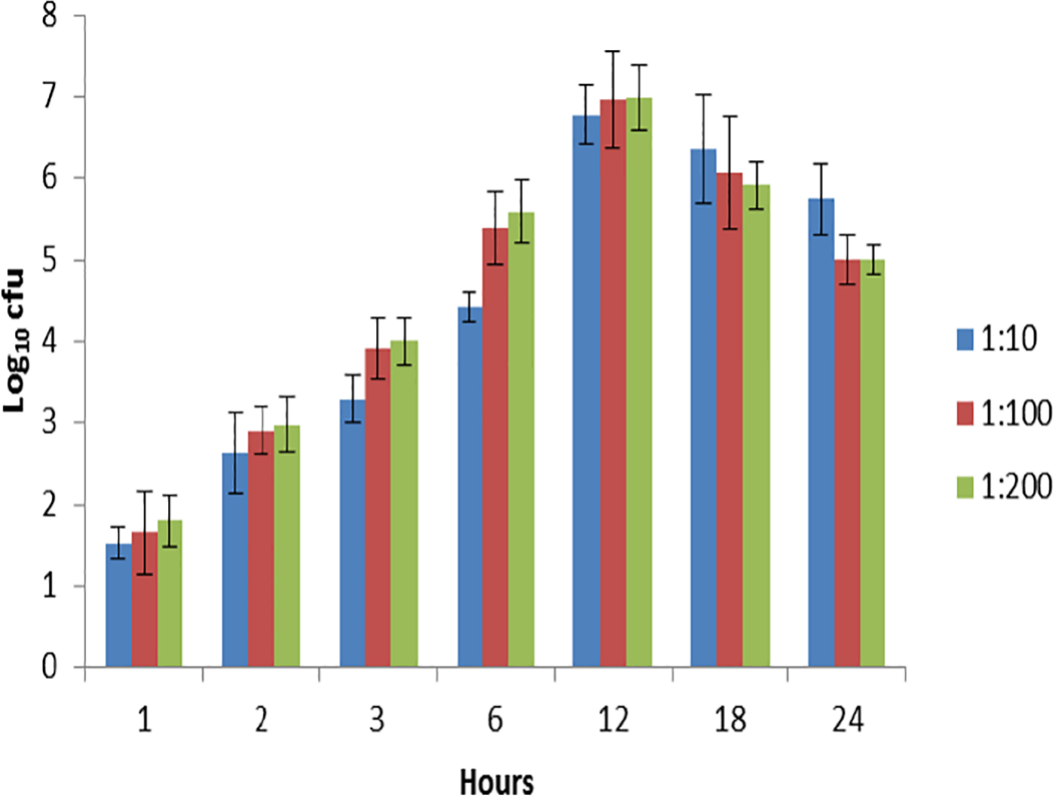
Intracellular survival and replication assay. Intracellular survival and replication of *B*. *pseudomallei in A549 cell lines* were assayed at different time-points (1, 2, 3, 6, 12, 18, 24 h) following 2 hours post-infection with MOI of 1:10, 1:100 and 1:200. At each time-point, the cells were lysed and colony-forming units (CFU) per milliliter of bacteria recovered were determined. Values indicate mean±standard error of 3 independent experiments assayed in triplicate.

The ability of *B*. *pseudomallei* to survive and replicate intracellularly demonstrated an increase from 1-12h post-infection, although a slight decrease was observed between 18 and 24h post-infection (Fig 1). No significant differences were observed in the intracellular survival and replication abilities at 1 and 2h post-infection among all the three MOIs used. However, at 3 and 6h post-infection there was a significant (p<0.05) difference between the MOI 1:10 (log_10_ cfu of 3.29 at 3h and 4.42 at 6h post-infection) as compared to the MOI 1:100 (log_10_ cfu of 3.91 at 3h and 5.39 at 6h post-infection) and 1:200 (log_10_ cfu of 4.00 at 3h and 5.59 at 6h post-infection). At 12h post-infection, the number of intracellular bacteria observed at all three MOIs was almost the same with the log_10_ cfu values of 6.78, 6.95 and 6.99 at MOIs 1:10, 1:100 and 1:200, respectively.

### Live *B*. *pseudomallei* and secretory proteins induce alterations in the gene transcription of A549 cells

**Fig 2:**
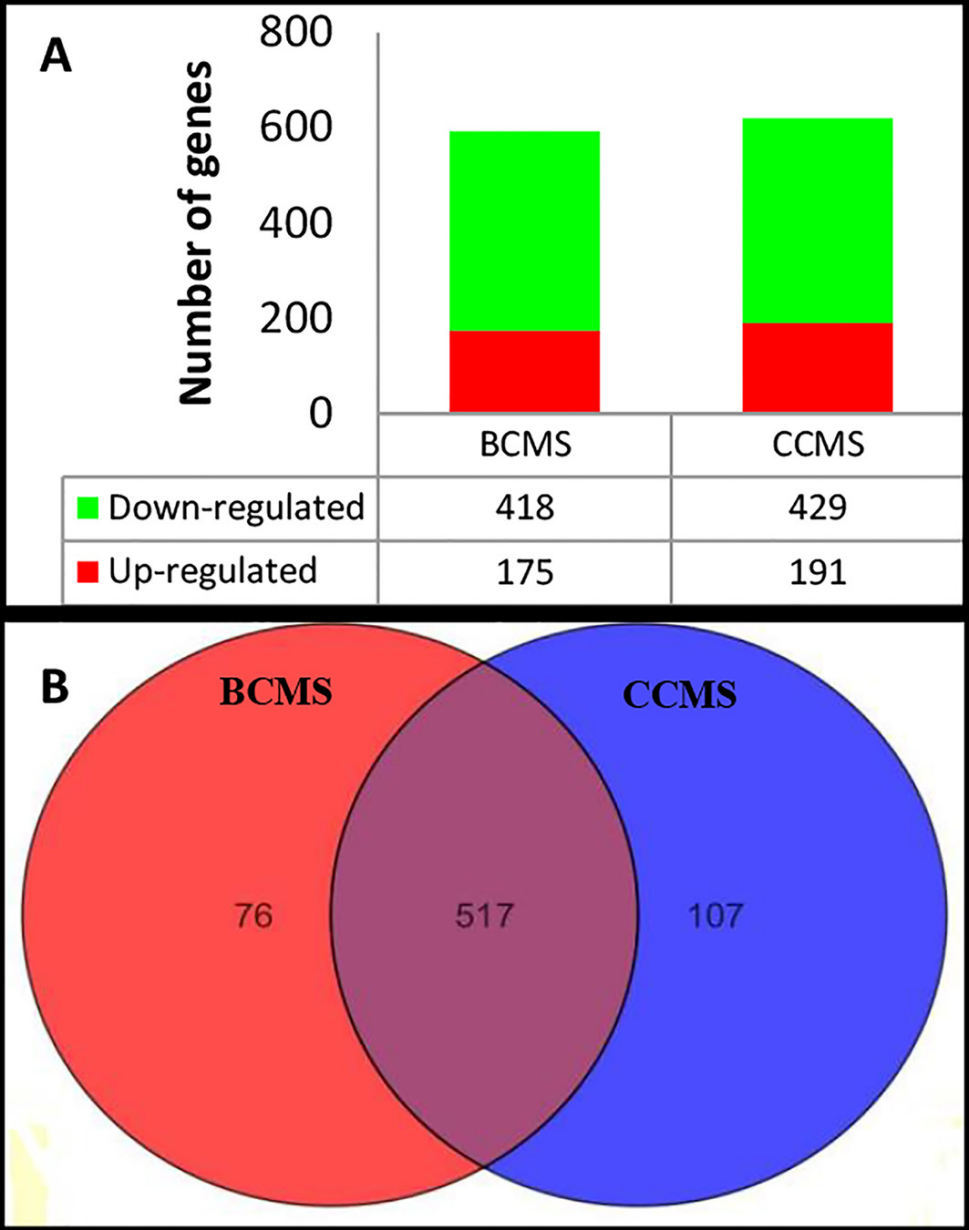
Differential expression of genes under the BCMS and CCMS conditions. (A) Number of genes that were significantly (p<0.05; fold change ≥2) up-regulated and down-regulated. (B) Venn diagram of the number of genes commonly regulated both by the BCMS and CCMS conditions, and the number of genes regulated exclusively by each of the conditions.

Of the 47323 total genes, 32339 (68.34%) that only have the Present and Marginal cut-off in at least one sample was filtered and selected. Using One-way ANOVA with Benjamini Hochberg (multiple testing) corrections, 2560 of the 32339 genes with Present and Marginal cut-off were filtered and identified as significantly expressed (p<0.05). The 2560 significantly expressed genes were further analyzed using the Volcano plot, which allows statistical and fold change analyses between two conditions. Exposure to live bacteria (BCMS) was found to differentially regulate 593 genes and the exposure to secretory proteins (CCMS) differentially regulated 624 genes as compared to uninfected control cells with a cut-off of 2-fold and a p-value of <0.05 (Fig 2A).

In general, under both BCMS and CCMS conditions, the number of down-regulated genes outnumbered the up-regulated genes. Of the 593 genes that were differentially regulated in the BCMS condition, 418 genes were found to be down-regulated and 175 genes up-regulated. Of the 624 differentially regulated genes under the CCMS condition, 429 were down-regulated and 191 were up-regulated. Further analysis using Venn diagrams revealed the presence of 517 genes that were commonly regulated under both the BCMS and CCMS conditions, whereas, 76 and 107 genes were exclusively regulated by the BCMS and CCMS conditions, respectively (Fig 2B).

### Live *B*. *pseudomallei* and secretory proteins influence the differential expression of molecules associated with metabolic pathways of A549 cells

Using GeneTrail, we found that the secretory proteins were found to regulate numerous cellular pathways (Table 3). However, both live *B*. *pseudomallei* and secretory proteins significantly (p<0.05) up-regulated pathways associated with metabolism, especially involving starch and sucrose, ascorbate and aldarate, and pentose and glucuronate interconversions. Similarly, pathways associated with cell signalling (neutrophin signalling, insulin signalling, TGF-β signalling and Hedgehog signalling) and cell adhesion (focal adhesion and cell adhesion molecules (CAMs)) were also up-regulated. Secretory proteins also exclusively regulated other signalling pathways (adipocytokine signalling, FcεRI signalling, JAK-STAT signalling, ErbB signalling, chemokine signalling and mTOR signalling), tight junction, FcγR-mediated phagocytosis and apoptosis signalling pathways.

**Table 3:** KEGG pathways significantly regulated by live *B*. *pseudomallei* bacteria and its secretory proteins (using GeneTrail and KEGG pathway mapper).

Pathway regulation	KEGG pathway	BCMS		CCMS	
		p-value	Genes involved	p-value	Genes involved
**Up- regulated**	Starch and sucrose metabolism	1.87e^-4^	G6PC2; UGT1A7; UGT1A9; UGT1A10	5.47e^-3^	G6PC2; UGT1A7; UGT1A9
	Ascorbate and aldarate metabolism	3.68e^-4^	UGT1A7; UGT1A9; UGT1A10;	1.34e^-2^	UGT1A7; UGT1A9
	Pentose and glucuronate interconversions	5.11e^-4^	UGT1A7; UGT1A9; UGT1A10;	1.65e^-2^	UGT1A7; UGT1A9
	Neutrophin signalling pathway	4.82e^-3^	AKT2; BCL2; NTRK3; SHC1	1.52e^-3^	AKT2; BCL2; NTRK3; SHC1; AKT3
	Insulin signalling pathway	6.49e^-3^	AKT2; G6PC2; PDE3B; SHC1	2.89e^-4^	AKT2; G6PC2; PDE3B; SHC1; SOCS2; AKT3
	Metabolism of xenobiotics by cytochrome P450	6.84e^-3^	UGT1A10; UGT1A7; UGT1A9	-	-
	TGF-β signalling pathway	1.12e^-2^	BMP2; BMP5; FST	1.99e^-2^	BMP2; BMP5; FST
	Focal adhesion	2.38e^-2^	AKT2; BCL2; COL4A1; SHC1	1.12e^-2^	AKT2; BCL2; COL4A1; SHC1; AKT3
	Cell adhesion molecules (CAMs)	3.78e^-2^	CDH4; NRCAM; VCAN	1.30e^-2^	CDH4; NRCAM; VCAN; JAM3;
	Hedgehog signalling pathway	3.81e^-2^	BMP2; BMP5	6.38e^-3^	BMP2; BMP5; ZIC2
	ErbB signalling pathway	-	-	2.11e^-2^	AKT2; AKT3; SHC1
	Chemokine signalling pathway	-	-	3.89e^-2^	AKT2; AKT3; DOCK2; SHC1
	mTOR signalling pathway	-	-	4.88e^-2^	AKT2; AKT3
	Apoptosis	-	-	2.17e^-2^	AKT2 AKT3 BCL2
	Fc gamma R-mediated phagocytosis	-	-	3.79e^-3^	AKT2; AKT3; DOCK2; PRKCE
	Adipocytokine signalling pathway	-	-	1.05e^-2^	AKT2; AKT3; G6PC2
	Fc epsilon RI signalling pathway	-	-	1.63e^-2^	AKT2; AKT3; PRKCE
	JAK-STAT signalling pathway	-	-	2.06e^-2^	AKT2; AKT3; IL11; SOCS2
	Tight junction	-	-	2.00e^-3^	AKT2; AKT3; EPB41L2; JAM3; PRKCE
**Down-regulated**	Complement and coagulation cascades	2.87e^-3^	C3; C4BPB; C5; CFB; CFH; F3	7.70e^-4^	C3; C4BPB; C5; CFB; CFH; F3; C1S;
	Drug metabolism–cytochrome P450	3.82e^-3^	ALDH1A3; AOX1; GSTM2; GSTM3; MAOA; UGT2B11	5.35e^-3^	ALDH1A3; AOX1; GSTM2; GSTM3; MAOA; UGT2B11
	Arginine and proline metabolism	4.92e^-3^	ASS1; CKB; GATM; MAOA; GLS;	2.78e^-2^	ASS1; CKB; GATM; MAOA
	Nicotinate and nicotinamide metabolism	1.28e^-2^	AOX1; CD38; NNT	1.54e^-2^	AOX1; CD38; NNT
	Glutathione metabolism	1.94e^-2^	ANPEP; GPX2; GSTM2; GSTM3	2.43e^-2^	ANPEP; GPX2; GSTM2; GSTM3
	Alanine, aspartate and glutamate metabolism	2.78e^-2^	ADSSL1; ASS1; GLS	-	-
	Lysosome	3.89e^-2^	CTSB; CTSF; GAA; HYAL1; IDUA; MAN2B1	1.75e^-2^	CTSB; CTSF; GAA; HYAL1; MAN2B1; CTSH; CTSS
	Phagosome	4.49e^-2^	C3; CD14; HLA-DMA; ITGB2; OLR1; SCARB1; TUBB2B	8.14e^-3^	C3; CD14; HLA-DMA; ITGB2; OLR1; SCARB1; TUBB2B; CTSS; THBS1
	Antigen processing and presentation	-	-	7.38e^-3^	CTSB; CTSS; HLA-DMA; HSPA2; KIR2DL1; KLRC3
	ECM-receptor interaction	-	-	1.05e^-2^	COL5A2; ITGB4; LAMB2; LAMB3; LAMC3; THBS1
	PPAR signalling pathway	-	-	1.80e^-2^	CPT1C; FADS2; OLR1; SLC27A1; SLC27A2
	Histidine metabolism	-	-	2.56e^-2^	ALDH1A3; AMDHD1; MAOA

Both live *B*. *pseudomallei* and secretory proteins also showed similar significant down-regulation (p<0.05) of metabolic pathways (drug metabolism–cytochrome P450, arginine and proline metabolism, nicotinate and nicotinamide metabolism, glutathione metabolism) and pathways associated with complement and coagulation cascades, lysosome and phagosome. Secretory proteins also exclusively regulated the pathways associated with antigen processing and presentation, ECM-receptor interaction and PPAR signalling.

Use of GATHER with the activated functional network inference component revealed significant associations with more pathways in KEGG, including the up-regulation of apoptosis and MAPK signalling pathways in BCMS, and MAPK signalling pathway with the CCMS conditions (Table 4). Similar down-regulation of cytokine-cytokine receptor interaction, JAK-STAT signalling, oxidative phosphorylation and Toll-like receptor (TLR) signalling pathways were identified both under the BCMS and CCMS conditions.

**Table 4:** KEGG pathways significantly regulated by live *B*. *pseudomallei* and secretory proteins (using GATHER with the activated Infer from Network component).

Condition	Regulation	KEGG pathway	Number of genes	p-value	Bayes factor
**BCMS**	Up-regulated	Apotosis	38	0.003	23
		MAPK signalling pathway	45	0.05	3
	Down-regulated	Cytokine-cytokine receptor interactions	111	0.001	38
		Jak-STAT signalling pathway	59	0.01	11
		Toll-like receptor signalling	40	0.02	8
**CCMS**	Up-regulated	MAPK signalling pathway	46	0.05	3
	Down-regulated	Cytokine-cytokine receptor interactions	110	0.001	35
		Jak-STAT signalling pathway	59	0.02	10
		Toll-like receptor signalling	40	0.02	8

### Infection of A549 cells by *B*. *pseudomallei* and exposure to bacteria-derived secretory proteins leads to regulation of genes associated with cellular metabolism

**Fig 3:**
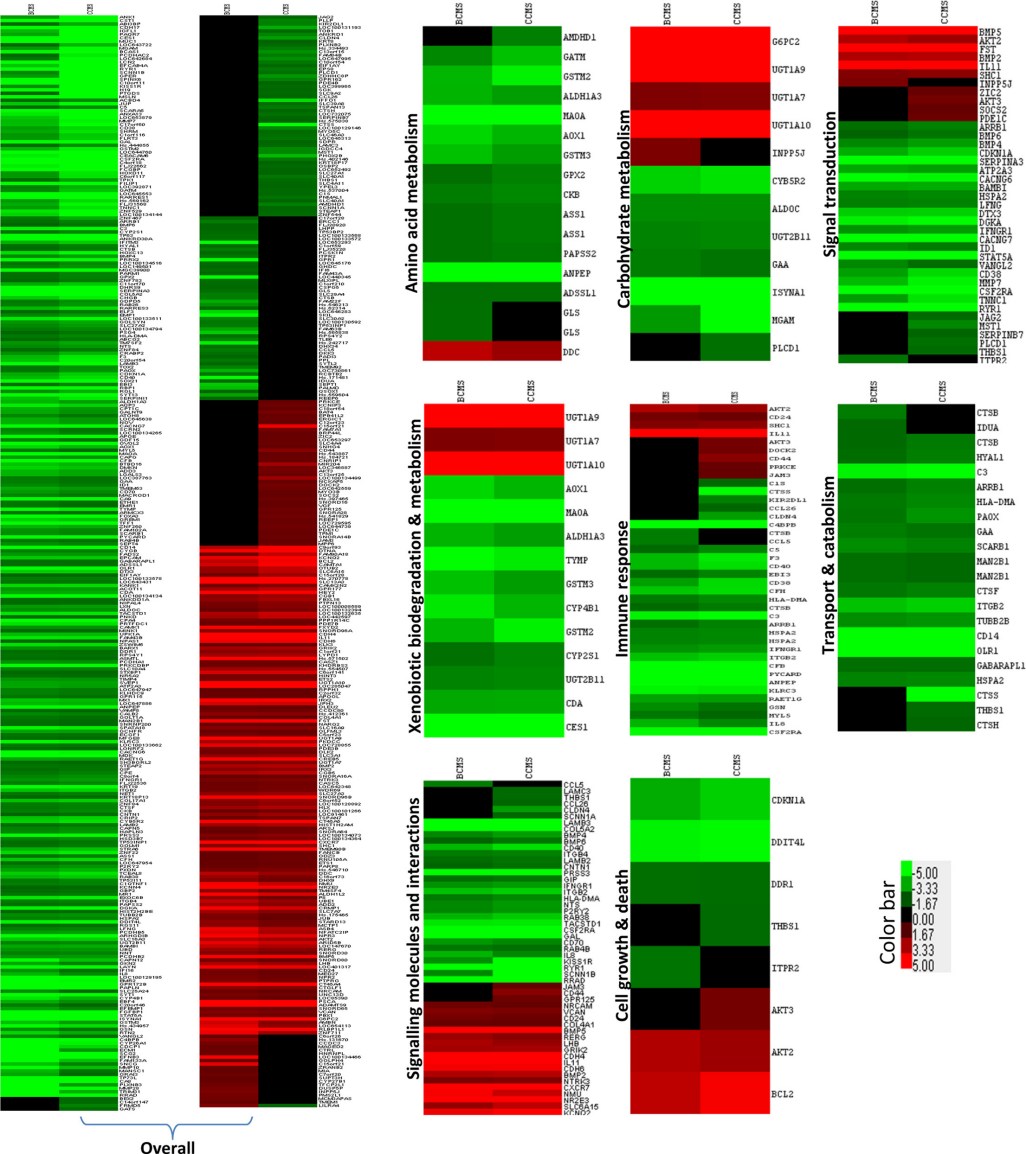
Heat map analysis of host (A549, human lung epithelial cell) transcriptional responses to early exposure with live *B*. *pseudomallei* and its secretory proteins. Hierarchical clustering of the expression profile is grouped according to functional categories. Heat maps indicate the fold change in A549 cells gene expression >2-fold (red) or <2-fold (green). Genes with no expression change are colored in black.

Both the BCMS and CCMS conditions were found to up-regulate several host genes involved in carbohydrate metabolism, a basic metabolic process that provides carbon and energy (Fig 3). Both conditions were found to highly up-regulate *G6PC2*, which encodes glucose-6-phosphatase; demonstrating 13.2 and 6.6 fold up-regulation by BCMS and CCMS, respectively. Further, *uridinediphospho-glucuronosyltransferase* (*UDPGT*), which is important in the conjugation and subsequent elimination of potentially toxic xenobiotics and endogenous compounds including UDPGT 1 family, polypeptide A10, A7 and A9 (*UGT1A10*, *UGT1A7*, *UGT1A9*), were also up-regulated.

Down-regulation of genes associated with amino acid metabolism that likely indicate amino acid starvation due to host membrane damage was also observed under both the BCMS and CCMS conditions (Fig 3). Host genes, including *argininosuccinate synthase* (*ASS1*), *creatine kinase (CKB)*, *glycine amidinotransferase (GATM)*, *glutaminase (GLS)*, *monoamine oxidase (MAOA)* and *adenylosuccinate synthase (ADSSL1)* were down-regulated under the BCMS condition. In addition to MAOA, ASS1, CKB, and *GATM*, the CCMS condition also down-regulated other genes involved in amino acid metabolism including *alanyl aminopeptidase* (*ANPEP*), *glutathione peroxidase* (*GPX2*), *glutathione S-transferase* (*GSTM2*, *GSTM3*), *aldehyde dehydrogenase (ALDH1A3)*, and *amidohydrolase (AMDHD1)*.

### Infection of A549 cells by *B*. *pseudomallei* and exposure to bacteria-derived secretory proteins results in broad down-regulation of host defense genes

Generally, genes associated with the immune system were down-regulated under both BCMS and CCMS conditions. Under BCMS, complement components (*C3* and *C5*), *C4-binding protein (C4bPB)*, *complement factor (CFB and CFH)*, and *coagulation factor (CF3)* were down-regulated. Down-regulations of *chemokine ligand 5 (CCL5)*, *CD14*, *CD40*, *TNF receptor* and *interleukin 8 (IL-8)* were also observed reflecting the likely evasion of *B*. *pseudomallei* of the TLR signalling and innate immune functions in the host. Additionally, genes involved in the lysosome pathway including *cathepsin (CTSB* and *CTSF)*, *glucosidase (GAA)*, *hyaluronoglucosaminidase (HYAL1)*, *iduronidase (IDUA)* and *mannosidase (MAN2B1)*, and genes associated with the phagosome pathway including *C3*, *CD14*, *major histocompatibility complex (HLA-DMA)*, *integrin (ITGB2)*, *oxidized low density lipoprotein (OLR1)*, *scavenger receptor (SCARB1)* and *tubulin (TUBB2B)* were also significantly down-regulated.

### Infection of A549 cells by *B*. *pseudomallei* and exposure to bacteria-derived secretory proteins leads to altered expression of genes associated with cellular signal transduction

Several genes associated with cell communication and signalling molecules were also up-regulated in response to live *B*. *pseudomallei* and secretory proteins. Both BCMS and CCMS conditions were found to up-regulate *bone morphogenetic protein (BMP2* and *BMP5)* and *follistatin (FST)* reflecting the up-regulation of TGF-β signalling pathway. Similarly, activation of serine/threonine-protein kinases (*Akt2* and *Akt3*) in combination with B-cell CLL/lymphoma (*Bcl2*), collagen (*COL4A1*), Src transforming protein (*SHC1*) lead to the activation of focal adhesion pathway. The cell adhesion molecule (CAMs) pathway was also activated under both BCMS and CCMS conditions with the up-regulation of *cadherin* (*CDH4*), neuronal cell adhesion molecule (*NRCAM*), a member of the Ig superfamily and versican (VCAN). In addition, CCMS also up-regulated the junctional adhesion molecule, *JAM3* that serves as a counter-receptor for integrin and alpha M (complement component 3 receptor 3 subunit) (ITGAM) that are involved in the regulation of transepithelial migration of polymorphonuclear neutrophils (PMNs).

### Infection of A549 cells by *B*. *pseudomallei* and exposure to bacteria-derived secretory proteins results in altered expression of genes associated with cellular proliferation and survival

The ErbB and mTOR signalling pathways were also up-regulated under the CCMS condition with the up-regulation of *Akt2*, *Akt3* and *SHC1*. Coupling of binding of extracellular growth factor ligands to intracellular signalling pathways by the ErbB family of receptor tyrosine kinases (RTKs) regulates diverse biologic responses, including proliferation, differentiation, cell motility, and survival. Finally, we also validated the microarray data obtained from the investigations. The qRT-PCR performed on the same samples used for microarray analysis confirmed the microarray results obtained. The genes analysed were confirmed as up-regulated or down-regulated in correlation with results obtained in the microarray analysis, indicating that the trend was comparable between both techniques used. However, the magnitude of regulation obtained by qRT-PCR was different compared to that with microarray analysis (Table 5).

**Table 5:** Validated genes regulated in response to live *B*. *pseudomallei* and its secreted proteins.

Symbol	Description	BCMS		CCMS	
		Array	qRT-PCR	Array	qRT-PCR
*G6PC2*	Glucose-6-phosphatase, catalytic, 2	13.24	16.33	6.57	11.26
*CES1*	Carboxylesterase 1	-14.14	-10.10	-24.44	-18.74
*CXCR7*	Chemokine (C-X-C motif) receptor 7	5.78	8.34	6.23	8.99
*LAYN*	Layilin	-13.80	-15.2	-10.65	-12.59
*SERPINA3*	Serpin peptidase inhibitor, clade A (alpha-1 antiproteinase, antitrypsin), member 3	-10.17	-9.67	-12.07	-11.73
*PYCARD*	PYD and CARD domain containing	-62.52	-79.98	-58.18	-85.77
*FXYD2*	FXYD domain containing ion transport regulator 2	41.36	65.13	41.63	68.52
*FST*	Follistatin	34.02	35.11	33.22	35.00

## Discussion

Different *Burkholderia* spp. appear to interact distinctly with host cells as evident from *in vitro* experiments [23, 24], and show marked variation in virulence attributes as seen from murine experiments [25]. Thus, in view of strain dependent variations, the invasive and intracellular survival abilities of the *B*. *pseudomallei* isolate used in this study, in A549 cells, was studied prior to the host response investigations. Lung epithelial cells have been shown to defend infections resulting from inhalation as they are amongst the first cells coming in contact with *B*. *pseudomallei* [26–28]. Hence, utilization of the A549 cells in an *in vitro* model may provide insights into pathogenesis events that likely occur in pulmonary melioidosis [29]. Besides, A549 cells have also been widely used as cell models in many experimental intracellular infections [30] and host transcriptional studies [27, 31, 32]

Here, we showed that *B*. *pseudomallei* strain was able to invade, survive and replicate intracellularly in the A549 cells; and the percentage of invasion, intracellular survival and replication abilities correlated with the set MOIs for up to 12 h. However, decrease in the percentage of intracellular bacteria relative to the initial inoculum was observed, at all the MOIs used, post-12h of contact with A549 cells, which partly could be attributed to the higher reduction in viability of the A549 cells post-12h of infection. Interestingly, the percentage of invasion at 2hr post infection was found to be very low (0.002–0.004%) compared to other studies that have reported higher percentage of invasion (0.1–0.9%) in the same duration [7, 33]. However, using similar method and MOI, our group has demonstrated higher percentage (~0.6% and >1.0%) of invasion of different *B*. *pseudomallei* strains [34, 35]. Thus, we strongly believe that the low invasion efficiency observed may likely be a strain dependent variation. As such, in this study, the host (A549 cells) responses to both *the* live *B*. *pseudomallei* (BCMS) and its secreted proteins (CCMS) was elucidated following three hours of exposure, when the invasion efficiency is comparably higher (~0.4%). Both the actively dividing bacteria and the stationary phase secreted proteins were used since it may provide an advantage to gain a complete picture of the host responses to different virulence factors.

Successful intracellular pathogens cause perturbation to host cellular functions and interfere with various metabolic, immune response and signalling pathways [36]. Here, we have demonstrated that several host carbohydrate metabolic pathways were significantly up-regulated in response to *B*. *pseudomallei* and its secreted proteins. Similar up-regulation of metabolic pathways in human airway cells upon exposure to live bacteria and soluble factors of other pathogens has been previously reported [21, 32, 37]. As an intracellular pathogen, *B*. *pseudomallei* is able to influence host cell activities prior to invasion via the secreted products, adhesins or the effector proteins of the type III or VI secretion systems injected into host cells, as well as upon internalisation [38]. By triggering the host cell metabolism, the bacteria may gain proliferation advantage as they depend on the energy sources and metabolites imported from or produced by the host cells, for respiration and cell division [39, 40]. However, in the host, metabolic regulations have previously been linked to other cell responses occuring during infection, especially regulation of pathways related to cell transformation, inflammation, and specific immune response [36]. Thus, the up-regulation of host metabolic pathways during early infection suggests the likely utilization of host resources by the bacteria as a means of adaptation and intracellular survival, as well as contributing to the maintenance of homeostasis in the host system; providing survival benefits for both pathogen and the host [21].

We have also found that the host amino acid metabolism was down-regulated in both the experimental conditions, possibly due to host membrane damage. Similarly, Tattoli *et al*. (2012) observed intracellular amino acid starvation during *Salmonella* and *Shigella* infections, attributed to host membrane damage [41]. It was also suggested that pathogen-induced amino acid starvation could downplay the activity of mTOR subsequently leading to induction of anti-bacterial autophagy. However, the mTOR pathway is known to be activated in phagocytes in response to bacterial infection or exposure to LPS [42]. Of note, the up-regulation of mTOR was observed only in the CCMS and not in the BCMS condition. This may be attributed to the short exposure time (three hours) with *B*. *pseudomallei*. However, the proteins likely activate the mTOR pathway during the same duration of exposure due to the concentrated factors in contact with A549 cells.

Down-regulation of several complement and coagulation cascades under both the experimental conditions was also observed. Extracellular polysaccharide capsule of *B*. *pseudomallei* reportedly down-regulates C3b consequently interfering with the activation of the alternate complement pathway [43]. The observed suppression of the alternate complement and coagulation pathways likely increases the chances of bacteria to evade the innate immune responses. In addition, *B*. *pseudomallei* and its secreted proteins were also found to suppress the lysosome and phagosome pathways. This may prevent the bacteria from being digested within the phagolysosome, resulting in early escape of the bacteria from lysosomal defensins and free radicals [44].

The anti-inflammatory cytokine IL-11 was highly up-regulated (~6.5 folds) by *B*. *pseudomallei*. Others have detected significantly higher levels of IL-11 in *C*. *trachomatis*-infected cultures of polarized HeLa cells. Of note, the level of IL-11 was significantly higher in infection involving a disseminating serovar relative to a non-disseminating variant [45]. The immunosuppressive role of IL-11 allows the bacteria to escape from host innate defenses for better dissemination. Gan (2005) reviewed that BALB/c mice infected with *B*. *pseudomallei* died of septicemia a few days after infection [46], suggestive of failure of the host innate immune response to clear the pathogen. Based on our current findings, we questioned if *B*. *pseudomallei* has the ability to silence the host innate defenses and/or the adaptive immune responses.

Bacteria are known to employ mechanisms to downplay the host immune system to ease intracellular entry. In this study, MX1 (myxovirus resistance protein), an important intrinsic host restriction factor, was down-regulated by *B*. *pseudomallei*. Nevertheless, others have also shown that MX1 is down-regulated in neutrophils exposed to *B*. *cepacia* [47]. Here, the down-regulation of *Mx1* is further supported by the down-regulation of neutrophil chemotactic factor IL-8, which plays an important role in the recruitment and activation of neutrophils during acute inflammation [48]. The down-regulation of IL-8 could likely be an evasion strategy put forth by *B*. *pseudomallei* to evade from inflammation [48]. Blockade of IL-8 binding to IL-8R1 curtails neutrophil infiltration at the site of pathogen entry leading to masking of inflammation. Hence, it is possible that *B*. *pseudomallei* evade the innate responses to favour intracellular establishment in the host, partly by silencing the recruitment of inflammatory mediators at the site of bacterial entry. Down-regulation of C3, a key immune glycoprotein factor was also observed. C3 deficiency is known to increase the susceptibility of host cells to invasive bacterial infections. Similarly, *Yersinia enterocolitica*, *an* intracellular pathogen, has been shown to mediate complement evasion by inactivating C3 factor following entry. The most interesting finding is the up-regulation of suppressor of cytokine signalling 2 (SOCS2), a well-known immunosuppressor [49], following exposure of the cells to supernatants. However, the STAT signalling pathway whereby SOCS2 operates still remains a grey area of investigation. The down-regulation of CD40 points to the potential suppression of adaptive immune responses following bacterial entry into the cytosol. The down-regulation of CD40 following exposure to *B*. *pseudomallei* is important as it is a likely prelude to compromising the initiation of adaptive responses against pathogens. Hence, it is likely that *B*. *pseudomallei* could harness the onset of negative modulation of the immune system early during infection. Hence, we postulate the likely role of *B*. *pseudomallei* in compromising the adaptive immune system.

During an infection, pathogens aim to establish a replicative niche to survive and multiply in the host. In line with this, several studies have demonstrated the prevention of host apoptosis as a mechanism of immune evasion and provide survival advantage by allowing the bacteria to replicate within the host [30, 50–53]. Conversely, we found that early exposure of *B*. *pseudomallei* and its secretory proteins to A549 cells significantly up-regulated the mediators of cellular apoptosis. Many microbial virulence factors have been shown to promote host apoptosis [54]. Additionally, *ERCC1*, one of the key molecules involved in DNA replication and repair, was down-regulated leading to DNA damage, which may act as a signal for apoptosis [55]. Alterations were also observed in the gene expression associated with cell cycle progression or arrest, notably *SPATA18* that was significantly down-regulated. However, *Bcl2*, important for preventing permeability of the mitochondrial membrane, was significantly up-regulated. Faherty and Maurelli (2008) have reported that the up-regulation of *Bcl2* enhances the pro-survival state of a cell [56]. Other regulators of survival, including *Akt2* and *Akt3* were also significantly up-regulated, supporting the notion of ongoing cell survival [57]. The changes observed, both in expression of genes associated with apoptosis and cell cycle progression, likely reflects the complex bacteria-host interplay. It is postulated that intracellular pathogens rely on the survival of host cell. As such, continuous division of the eukaryotic cells is important for prolonged bacterial survival in the host [56]. Hence, the balance between these processes likely determines whether the cell should survive or undergo apoptosis.

I*n vitro* responses of human A549 cells to live *B*. *pseudomallei* and its secreted proteins revealed vital features that may be relevant to the initial stages of contact between the pathogen and the host. These responses suggest the efforts of host cells to recruit and activate various arms of the immune system, and contain local infection. At the same time, the pathogen strives to suppress the host immune responses, disseminate and continue to multiply or persist. However, further functional analysis and validation of the results presented coupled with studies on the *transcriptional adaptation* of *B*. *pseudomallei* in the lung epithelial cells is warranted for comprehensive understanding of the strategies utilised to survive and cause disease. Further analyses of the molecular interaction between the bacteria and the host will also help provide fascinating insights to illuminate the complex interplay between the host and pathogen, and provide basis for the development of novel strategies for detecting and preventing invading pathogens.
